# Supplementary material for: Gynaecological Health Patterns and Motherhood Experiences of Female Professional Football Players
Source: Int J Environ Res Public Health. 2025 Jan 21;22(2):136. doi: 10.3390/ijerph22020136 (PMC11855395; doi:10.3390/ijerph22020136)
Supplement: Supplementary file 1 [file ijerph-22-00136-s001.zip › ijerph-3365455-supplementary.pdf]

Supplementary Materials:
www.mdpi.com/xxx/s1.

What is your age?
[Number]
How tall are you (cm)?
[Number]
How much do you weigh (kg)?
[Number]
What is your percentage of body fat (%)?
[Number]
What is the highest level of education you have completed?
[No school completed; Nursery/Elementary school; High school; Vocational/technical school; College, university or equivalent]
Apart from your professional football career, are you currently studying?
[Yes; No]
If yes, how many hours per week on average do you study?
Apart from your professional football career, do you have currently another paid job?
[Yes; No]
If yes, what other kind of (paid) work do you do?
If yes, how many hours per week on average do you work (apart from your professional football career)?
What is your position on the field?
[Goalkeeper; Defender; Midfield; Forward]
At what level do you play?
[Highest national level/league; Second highest national level/league; Other]
For how many seasons are you a professional footballer?
[Number]
In the past few years, were you admitted to an hospital?
If yes, why?
Have you been diagnosed with a mental health disorder (e.g., depression, anxiety, insomnia)?
[Yes; No; I don't know]
If yes, which mental health disorder(s)?
How many cigarettes (approx.) have you smoked in your entire life?
[Number]
How many days have you smoked cigarettes during the past 30 days?
[Number]

Have you been pregnant?
[Yes; No]
IF YES How many times have you been pregnant?
[Number]
IF YES How did you get pregnant?
[Intercourse; Insemination with partner's sperm; Insemination with sperm donation; I rather not tell]
How many miscarriages did you have?
[Number]
Body dissatisfaction (validated EDI-3 subscale)
I think that my stomach is too big.
I think that my thighs are too large.
I think that my stomach is just the right size.
I feel satisfied with the shape of my body.
I like the shape of my buttocks.
I think that my hips are too big.
I think that my thighs are just the right size.
I think that my buttocks are too large.
I think that my hips are just the right size.
[Always; Usually; Often; Sometimes; Rarely; Never]
Drive for thinness (validated EDI-3 subscale)

I eat sweets and carbohydrates without feeling nervous.
I think about dieting.
I feel extremely guilty after overeating.
I am terrified of gaining weight.
I exaggerate or magnify the importance of weight.
I am preoccupied with the desire to be thinner.
If I gain a pound, I worry that I will keep gaining.
[Always; Usually; Often; Sometimes: Rarely; Never]
At about what age did you begin to menstruate?
[Age]
What is the average duration of your menstrual cycle (i.e., average number of days between the first day of your menstrual cycles)?
[Number of days]
What is the average duration of your menstrual bleeding period?
[Number of days]
How would you describe your menstrual cycle?
[Regular; Irregular, Very irregular]
Since your first menstrual cycle, did your menstrual bleeding period stop for three or more consecutive months (for other reasons than hormonal contraception)?
[No; Yes]
Do you generally experience pain during your menstrual cycle?
[No; Yes, before a bleeding period; Yes, during a bleeding period; Yes, after a bleeding period]
Are you currently using contraceptives?
[No; Yes, cap; Yes, pill; Yes, condom; Yes, contraceptive implant; Yes, contraceptive injection; Yes, contraceptive patch; Yes, diaphragm; Yes, female condom; Yes, namely…]
Are you currently using hormone replacement?
[No; Yes]
Are you a mother?
[No; Yes]
If YES How many children do you have?
[Number]
If YES How did you give birth?
[Vaginal delivery; Caesarean delivery; Adoption]
If DELIVERY How long did it take before you returned to training?
Child 1 Child 2 Child X [Number of weeks]
If DELIVERY How long did it take before you returned to competitive matches?
[Number of weeks]
Have you ever had a stress fracture?
[No; Yes]
If YES please indicate when it occurred and what bone(s).
Have you even been told you have low bone density (e.g., osteopenia or osteoporosis)?
[No; Yes]

How many severe injuries in your LEFT hip have you had so far as professional footballer?
[Number]
How many severe injuries in your RIGHT hip have you had so far as professional footballer?
[Number]
How many severe injuries in your LEFT knee have you had so far as professional footballer?
[Number]
How many severe injuries in your RIGHT knee have you had so far as professional footballer?
[Number]
How many severe injuries in your LEFT ankle have you had so far as professional footballer?
[Number]
How many severe injuries in your RIGHT ankle have you had so far as professional footballer?
[Number]
